# Differentiation of flea communities infesting small mammals across selected habitats of the Baltic coast, central lowlands, and southern mountains of Poland

**DOI:** 10.1007/s00436-014-3817-9

**Published:** 2014-03-12

**Authors:** Krzysztof Kowalski, Urszula Eichert, Michał Bogdziewicz, Leszek Rychlik

**Affiliations:** Department of Systematic Zoology, Institute of Environmental Biology, Faculty of Biology, Adam Mickiewicz University, Umultowska 89, Poznań, 61-614 Poland

**Keywords:** Alpha and beta diversity, Biogeography, Fleas, Latitude, Small mammals, Poland

## Abstract

Only a few studies comparing flea composition on the coast and in the mountains have been conducted. We investigated differences in flea communities infesting small mammals in selected habitats in northern, central, and southern Poland. We predicted (1) a greater number of flea species in the southeastern Poland and a lower number in the north, (2) a greater number of flea species in fertile and wet habitats than in poor and arid habitats, and (3) a low similarity of flea species between flea communities in western and eastern Poland. We found a negative effect of increasing latitude on flea species richness. We suppose that the mountains providing a variety of environments and the limits of the geographic ranges of several flea subspecies in southeastern Poland result in a higher number of flea species. There was a positive effect of increasing wetness of habitat on flea species richness. We found a high diversity in flea species composition between western and eastern Poland (beta diversity = 11) and between central and eastern Poland (beta diversity = 12). Re-colonization of Poland by small mammals and their ectoparasites from different (western and eastern) refugees can affect on this high diversity of flea species.

## Introduction

Species composition of fleas is affected by a variety of abiotic (e.g., temperature, humidity, precipitation, and elevation or structure of the substrate) and biotic factors (e.g., host species, its age, sex, behavior, and habitat preferences) (Marshall 1981; Krasnov et al. 2007, 2010b; Pawelczyk et al. 2004). Hosts with similar habitat requirements and diet can be infected by similar or even identical flea species (Krasnov et al. 2006b; Klimpel et al. 2007). The composition of flea species on host species is determined not only by host-flea relations but also by host-habitat relations. Therefore, a habitat for fleas is not a particular host or a group of hosts but rather a particular host or a group of hosts inside a particular habitat (Krasnov et al. 2006b). In addition, flea species composition varies less (1) among populations of the same host species than among different host species and (2) among habitats of the same type than among different habitats (Poulin and Valtonen 2002; Krasnov et al. 2006b).

Social species living in large family groups, like many rodents, are characterized by a higher prevalence of parasites than less social species like shrews, which are more solitary (Rychlik 1998; Karbowiak et al. 2005; Oguge et al. 2009; Krasnov et al. 2010a). In a more social animal, contacts between individuals are much more frequent which promotes the exchange of fleas. Similarly, increased densities have the same effect as increasing the number of contacts between individuals in population. So, high densities of fleas should mirror high host densities (Rödl 1979; Laakkonen 2000).

Flea species composition depends on the depth and stability of the host’s burrow (Krasnov et al. 2006a). Deep and permanent burrows with a constant microclimate seem to be a better habitat for fleas than the shallow and temporary burrows. As a result, the hosts living in deep and permanent burrows may have higher number of flea species and higher prevalence than hosts living in ephemeral and ground burrows (Krasnov et al. 2004, 2010a).

This study aimed to find differences in the flea species composition of small mammals in selected habitats of the Baltic coast, the central lowlands, and mountains of Poland. Due to the biogeography rule, the biodiversity of plant and animal species decreases with increasing latitude (Rohde 1992; Rosenzweig 1992; Krasnov et al. 2004; Pavoine and Bonsall 2011). Additionally, due to the fact that in Poland we can find the limits of the geographic ranges of several flea species and subspecies (Skuratowicz 1967; Bartkowska 1973, 1977), we predicted that (1) a greater number of flea species will be noted in the southeastern Poland and a lower number in the north. Moreover, fauna of small mammals is usually richer in fertile and wet habitats (e.g., Aulak 1970). We thus predicted that (2) a greater number of flea species will be recorded in fertile and wet habitats and a lower number in poor and arid habitats. There are some proofs that after the last glaciation, Poland was re-colonized by small mammals and their ectoparasites from different (western and eastern) refugees (Michaux et al. 2004, 2005; Deffontaine et al. 2005; Nieberding et al. 2008). Therefore, we expected (3) a low similarity of flea species between flea communities in western and eastern Poland.

## Materials and methods

### Study area

**Fig. 1:**
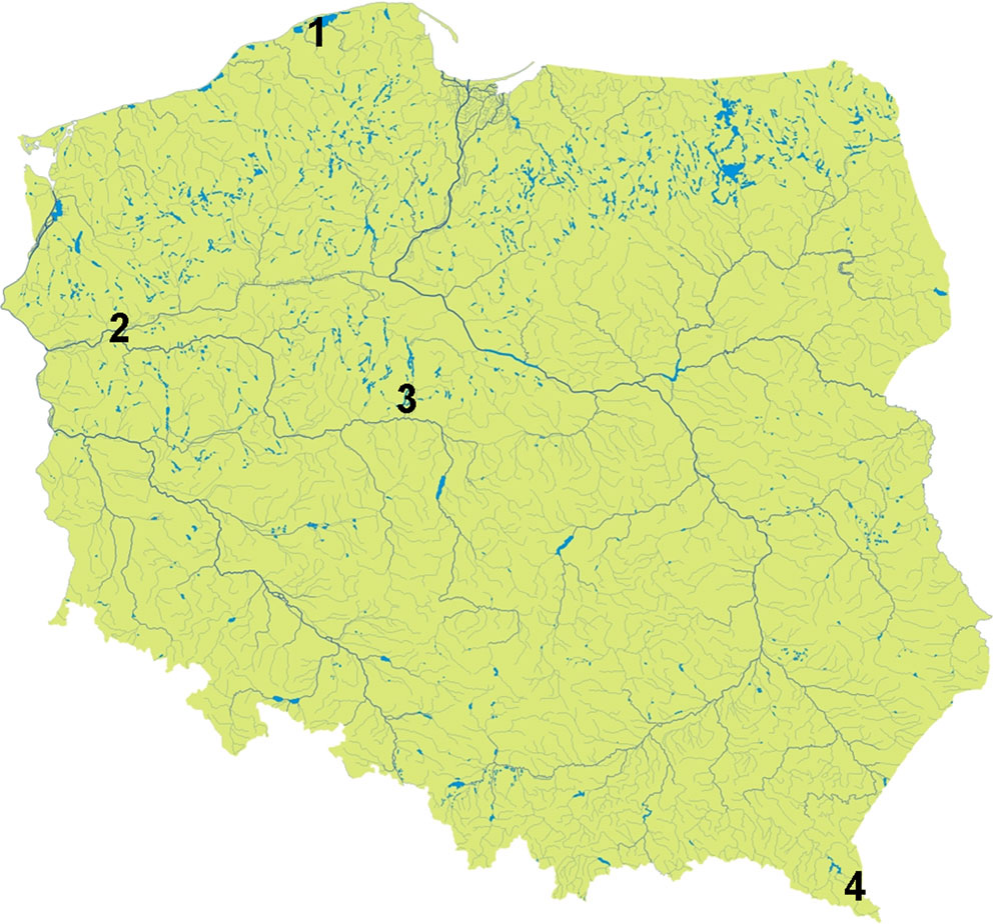
The location of the study plots: *1* Słowiński National Park, *2* Gorzowska Forest, *3* Konin lakes area, *4* Bieszczady Mountains

In total, 19 study plots were located in selected habitats of four regions: the Baltic coast (Słowiński National Park), the western lowlands (Gorzowska Forest), the central lowlands (Konin lakes area), and the mountains in the southeast (Bieszczady Mountains) of Poland (Fig. 1). In the Słowiński National Park (SNP), we established seven study plots. Plots S1–S4 were located in the central part of the park and were investigated for 2 weeks in late July and August 2010 (Table 4 in Appendix). Plots S5–S7 were located on the spit separating the Baltic Sea from the Gardno Lake (the western part of the park) and were studied in September 2011 (1 week). In the Gorzowska Forest, the study was conducted in July 2010 on two plots and in the Konin lakes area on eight plots from August to October 2011. In the Bieszczady Mountains, research was conducted for 5 days in August 2011 on two study plots near Lutowiska village.

### Trapping procedure

Small mammals were captured in wooden live traps, which were usually arranged in three parallel lines with 10 traps in each line (except S4, S7, G1, G2, and K4; see Table 4 in Appendix). The first line was located directly along the shoreline (traps were set within 1 m from water) or in the wettest habitat. The next two lines were set at distances about 10 and 20 m from the first line. The traps were spaced at approximately 10-m intervals within lines so it can be assumed that each study plot covered approximately 0.30 ha. Only in Gorzowska Forest traps were arranged in eight lines with eight traps in each line and covered 0.49 ha. We placed food (oat flakes and minced beef) in the traps as bait and as provision for captured animals. Trapping sessions were carried out at night for 5–10 days in the Słowiński National Park and for 3–4 days in the Bieszczady Mountains. Within the Konin lakes area, the study was carried out non-stop for 2 days. Traps were set in the afternoon and checked every 3–4 h in areas inhabited by shrews or every 5–6 h in areas inhabited only by rodents. In Gorzowska Forest, traps were active non-stop and checked twice a day for five consecutive days. The captured animals’ species were determined and they weighed. We recorded also their age, sex, and reproductive activity and individually marked by ear tagging (Gorzowska Forest) or cutting a small patch of fur (other locations). Then, mammals were placed in a canvas bag for 2–3 min to collect fleas (e.g., Haas and Walton 1973, Paramasvaran et al. 2009, Zuo et al. 2011). The collected fleas were placed in a vial with alcohol, and mammals were released at the place of capture.

### Data analysis

We analyzed the relationship between (1) the latitude and number of flea species and (2) between habitat richness and number of flea species using generalized linear mixed models implemented via “lme4” package (Bates et al. 2011). In both analyses, we used a number of flea species as response variable and location as random factor. In analysis (1) latitude, abundance of small mammals, number of small mammal species, and abundance of fleas were implemented as fixed factors, whereas in analysis (2) we used habitat wetness and habitat fertility, abundance of small mammals, number of small mammal species, and abundance of fleas as fixed factors. In both analyses we used Poisson error distribution, and we began with a model containing all the above mentioned explanatory variables and determined the structure of the final model through backward stepwise elimination of non-significant factors using likelihood ratio test. For the purpose of analysis (2), we categorized all habitats into five classes of wetness (1–least wet, 5–most wet) and five classes of fertility (1–least fertile, 5–most fertile). Habitats were assigned to fertility classes based on undergrowth density and the thickness of the litter and to wetness categories based on differences in plant communities. All the analyses were conducted in R software (R Development Core Team 2012).

We measured the alpha and beta diversity to find differences in species composition between flea communities in western and eastern Poland. Alpha diversity refers to the diversity within particular area (or ecosystem) and is usually expressed by the number of species (i.e., species richness) in that area. In turn, beta diversity refers to the total number of species that are unique to each of the areas (or ecosystems) being compared (Whittaker 1972, Harrison et al. 2004).

## Results

### Small mammals captured

In the Słowiński National Park, we captured 60 small mammals belonging to four rodent and three shrew species in 2010 and 34 mammals belonging to five rodent and two shrew species in 2011 (see Table 5 in Appendix). In the Konin lakes area, we captured 187 small mammals belonging to four rodent and two shrew species. In the Gorzowska Forest, we recorded 125 rodents belonging to two species, and in the Bieszczady Mountains, 75 small mammals belonging to five rodents and three shrews.

### Fleas collected

In total, on all study plots, we collected 634 individuals of fleas belonging to 17 species and subspecies. In the Słowiński National Park, we collected 18 individuals belonging to three species of fleas in 2010 and 65 fleas belonging to eight species in 2011 (Table 1). In the Gorzowska Forest, we found 294 fleas belonging to five species, and in the Bieszczady Mountains, we collected 110 fleas belonging to seven species and subspecies. In the Konin lakes area, we recorded 147 fleas belonging to 13 species (Table 2).

**Table 1:** Fleas collected from small mammals in the Słowiński National Park, Gorzowska Forest, and the Bieszczady Mountains

Area/Plot no.	Słowiński National Park												Gorzowska Forest				Bieszczady Mountains								
	S1	S2		S3			S5				S6	S7	G1		G2		B1			B2					
Flea species/ subspecies	Afl	Afl	Mgl	Afl	Agr	Moe	Mmn	Mgl	Mgr	Sar	Moe	Agr	Afl	Mgl	Afl	Mgl	Afl	Mgl	Sar	Smn	Sar	Agr	Afl	Mar	Mgr
*Ctenophthalmus agyrtes agyrtes*	4	2	1	1	1	–	–	–	4	–	–	–	39	52	24	11	–	–	–	–	–	–	–	–	–
*Ctenophthalmus agyrtes kleinschmidtianus*	–	–	–	–	–	–	–	–		–	–	–	–	–	–	–	2	2	–	–	1	5	27	–	3
*Ctenophthalmus bisoctodentatus*	–	–	–	–	–	–	–	–	–	–	–	–	–	–	–	–	–	–	–	–	–	–	1	–	–
*Ctenophthalmus solutus*	–	–	–	–	–	–	–	–	–	–	–	–	28	6	15	2	–	–	–	–	–	–	–	–	–
*Ctenophthalmus uncinatus*	–	–	–	–	–	–	–	–	–	–	–	–	4	47	–	6	1	1	–	–	–	–	–	1	–
*Doratopsylla dasycnema cuspis*	–	–	–	–	–	–	–	–	–	–	–	–	–	–	–	–	–	–	–	2	17	–	–	–	–
*Doratopsylla dasycnema dasycnema*	–	–	–	–	–	–	1	–	–	–	–	–	–	–	–	–	–	–	–	–	–	–	–	–	–
*Hystrichopsylla talpae orientalis*	–	–	–	–	–	–	–	–	–	–	–	–	–	–	–	–	–	–	1	–	–	–	–	–	–
*Hystrichopsylla talpae talpae*	–	–	–	–	–	–	–	–	8	–	2	–	–	–	–	–	–	–	–	–	–	–	–	–	–
*Megabothris turbidus*	–	–	1	–	–	1	–	3	9	–	–	1	25	27	3	4	2	10	–	–	–	2	8	–	–
*Megabothris walkeri*	–	–	–	–	–	7	–	–	23	–	–	–	–	–	–	–	–	–	–	–	–	–	–	–	–
*Palaeopsylla soricis*	–	–	–	–	–	–	–	–	–	1	–	–	–	–	1	–	–	–	2	3	18	–	–	1	–
*Peromyscopsylla bidentata*	–	–	–	–	–	–	–	–	3	–	–	–	–	–	–	–	–	–	–	–	–	–	–	–	–
*Peromyscopsylla silvatica*	–	–	–	–	–	–	–	–	10	–	–	–	–	–	–	–	–	–	–	–	–	–	–	–	–
Total	4	2	2	1	1	8	1	3	57	1	2	1	96	132	43	23	5	13	3	5	36	7	36	2	3

*Afl Apodemus flavicollis, Agr Apodemus agrarius,MarMicrotus arvalis, MglMyodes glareolus, MgrMicrotus agrestis, MmnMicromys minutus, MoeMicrotus oeconomus, Nfd Neomys fodiens, Sar Sorex araneus, Smn Sorex minutus*

**Table 2:** Fleas collected from small mammals in the Konin lake area. Mammal species codes as in Table 5

Plot no.	K1		K2	K3				K4	K5				K6			K7		K8
Flea species/subspecies	Agr	Mgl	Mgl	Agr	Afl	Mgl	Nfd	Afl	Agr	Afl	Asl	Mgl	Agr	Mgl	Sar	Afl	Sar	Agr
*Ctenophthalmus agyrtes agyrtes*	7	2	–	1	1	1	–	1	3	1	–	1	8	4	–	1	–	1
*Ctenophthalmus agyrtes peusianus*	–	–	–	–	–	–	–	–	–	–	–	–	–	1	–	–	–	–
*Ctenophthalmus assimilis*	–	–	–	–	–	–	–	–	–	–	–	–	–	–	1	–	–	–
*Ctenophthalmus bisoctodentatus*	2	–	–	–	–	–	–	–	–	–	–	–	–	–	–	–	–	–
*Ctenophthalmus solutus*	–	–	–	–	–	–	–	4	4	1	–	–	–	–	–	–	–	–
*Ctenophthalmus uncinatus*	1	1	1	–	–	1	–	–	–	–	–	–	–	–	–	–	–	–
*Doratopsylla dasycnema dasycnema*	–	–	–	–	–	–	–	–	–	–	–	–	–	–	–	–	1	1
*Hystrichopsylla talpae talpae*	–	–	–	–	–	1	–	1	1	–	–	–	1	–	–	–	2	–
*Megabothris turbidus*	20	20	2	1	2	8	1	2	1	2	–	3	4	–	–	1	–	1
*Megabothris walkeri*	–	–	–	–	–	–	–	–	1	–	–	–	–	–	–	–	–	–
*Nosopsyllus fasciatus*	–	–	–	–	–	–	–	–	5	–	2	–	9	–	–	–	–	–
*Palaeopsylla soricis*	–	–	–	–	–	1	–	–	–	–	–	–	–	–	2	–	1	–
*Peromyscopsylla bidentata*	–	–	–	–	–	–	–	–	–	–	–	–	–	1	–	–	–	–
Total	30	23	3	2	3	12	1	8	15	4	2	4	22	6	3	2	4	3

### Factors influencing diversity of fleas

**Fig. 2:**
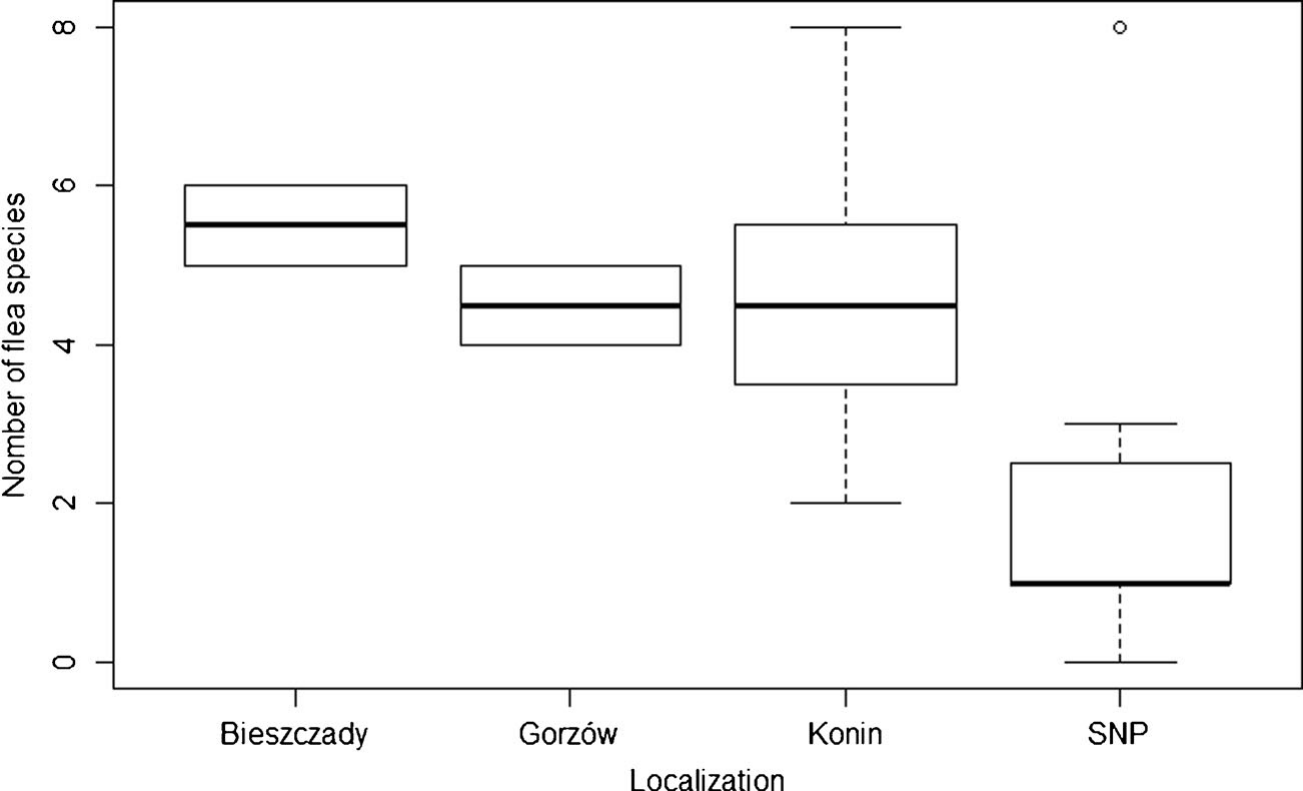
Number of flea species in different geographical localizations (with Bieszczady being outermost South and SNP farthest North). *Boxes* denote 25th, 50th, and 75th percentiles; *whiskers* represent the lowest and highest datum within the 1.5 interquartile range of the lower and upper quartile

**Fig. 3:**
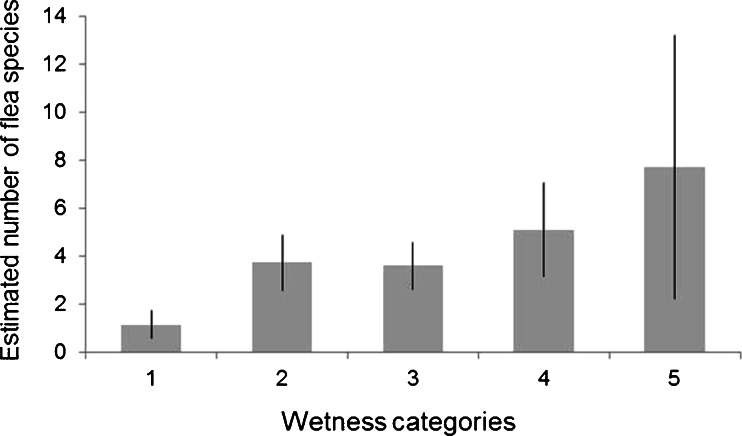
Number of flea species in habitats of different wetness categories. The differences between category 1 and categories 3 and 4 are significant (*p* < 0.05), and the difference between categories 1 and 2 is approaching significance (*p* = 0.08). *Whiskers* indicate standard errors

The final model in analysis (1) revealed in backward procedure included only latitude as an explanatory variable and the effect of increasing latitude on flea species richness was negative (*z*_4,18_ = −2.50, *p* = 0.01; Fig. 2). In the case of analysis (2), the final model included habitat wetness and habitat fertility as an explanatory variables. The differences in flea species number between habitats of different wetness categories were significant with increasing number of flea species along with the increasing humidity of habitat (Fig. 3). We found no pattern in relationship between number of flea species and habitat fertility.

The alpha diversity (species richness) for western Poland was 10, 13 for central, and 7 for eastern Poland. We found a low similarity of flea species/subspecies between western and eastern Poland (beta diversity = 11) and between central and eastern Poland (beta diversity = 12). The flea species composition of western and central Poland was more similar (beta diversity = 5; Table 3).

**Table 3:** Alpha and beta diversity for flea species/subspecies infesting small mammals in western (Słowiński National Park and Gorzowska Forest), central (Konin lakes area), and eastern (Bieszczady Mountains) Poland

Flea species/subspecies	Western Poland	Central Poland	Eastern Poland
*Ctenophthalmus agyrtes agyrtes*	x	x	
*Ctenophthalmus agyrtes kleinschmidtianus*			x
*Ctenophthalmus agyrtes peusianus*		x	
*Ctenophthalmus assimilis*		x	
*Ctenophthalmus bisoctodentatus*		x	x
*Ctenophthalmus solutus*	x	x	
*Ctenophthalmus uncinatus*	x	x	x
*Doratopsylla dasycnema cuspis*			x
*Doratopsylla dasycnema dasycnema*	x	x	
*Hystrichopsylla talpae orientalis*			x
*Hystrichopsylla talpae talpae*	x	x	
*Megabothris turbidus*	x	x	x
*Megabothris walkeri*	x	x	
*Nosopsyllus fasciatus*		x	
*Palaeopsylla soricis*	x	x	x
*Peromyscopsylla bidentata*	x	x	
*Peromyscopsylla silvatica*	x		
Alpha diversity	10	13	7
Beta diversity	Western vs. central Poland: 5	Central vs. eastern Poland: 12	Western vs. eastern Poland: 11

## Discussion

In accordance with our predictions, we recorded the inverse relationship between latitude and the number of flea species. In the same time, we found no effect of flea abundance, small mammal abundance, and number of small mammal species on number of flea species recorded. Overall, a well-known rule of biogeography is that the biodiversity of plant and animal species decreases with the increase of the distance from the equator (Rohde 1992; Rosenzweig 1992; Krasnov et al. 2004; Pavoine and Bonsall 2011). On the other hand, Krasnov et al. (2007) reported that the number of flea species in the region increases with the average elevation. This is likely not caused directly by the variation in altitude, but rather the presence of mountains, which presumably provide a variety of environments within the region, possibly resulting in a higher number of flea species. This relationship has been confirmed for Palaeoarctic realm (including Tatry Mountains), where they conducted their research, but have not been confirmed for Nearctic realm. Generally, this relationship is concerned for ectoparasites, which are susceptible to environmental factors that vary with the latitude (Krasnov et al. 2007). In contrast, endoparasites due to their stable habitat (inside the host body) do not show this relationship (Rohde and Heap 1998). Our studies were conducted in the Palearctic realm, and two study plots were located in the mountains. So, the high number of flea species in the Bieszczady Mountains can be explained by the availability of more diverse environments in this region. In addition, in Poland, we can find the northwestern limits of the geographic ranges of several flea species, which may also result in a high number of species in the southeastern part of the country (Bartkowska 1977). On the Baltic coast, the most abundant flea was *Megabothris walkeri* reaching the southern range in Poland. In the Bieszczady Mountains, we found two flea subspecies which reach their western geographic ranges in this region: *Hystrichopsylla talpae orientalis* and *Doratopsylla dasycnema cuspis*. Other authors also recorded these fleas in mountain areas (Skuratowicz 1967; Bartkowska 1973, 1977).

Nevertheless, the number of flea species not always is lower at higher latitudes. Krasnov et al. (2004) found a positive correlation between latitude and the number of flea species in the Palearctic realm. They suggest that the reason may be many species of mammals living in regions of temperate climate inhabit deeper and more permanent burrows than those of mammals from warmer regions, which in turn are more preferred by fleas (Krasnov et al. 2004, 2006a, 2010a) because they spend a large part of their life in the host nest. Our research was conducted only in Poland, i.e., only under temperate climate, so generally most small mammals living here can possess deeper burrows than mammals living in tropical regions. However, sandy grounds on the Baltic coast likely hinder digging deep burrows (Haitlinger 1972), which could result in observed lower number of flea species in the north.

There are some proofs that after the last glaciation, Poland was re-colonized by small mammals and their ectoparasites from different (western and eastern) refugees (Michaux et al. 2004, 2005; Deffontaine et al. 2005; Wójcik et al. 2010). Therefore, we expected differences in species composition between flea communities in western and eastern Poland. The results of our study confirmed our predictions. We found a low similarity between western and eastern Poland (11 species and subspecies). In the Bieszczady Mountains, we found four flea species/subspecies (*H. talpae orientalis*, *Ctenophthalmus agyrtes kleinschmidtianus*, *Ctenophthalmus bisoctodentatus*, and *Doratopsylla dasycnema cuspis*) which were absent in western Poland. *Hystrichopsylla talpae orientalis*, *D. dasycnema cuspis*, and *C. agyrtes kleinschmidtianus* reach their western geographic ranges in this region of Poland. In turn, seven flea species/subspecies were found only in western Poland. We found *Peromyscopsylla silvatica* only in western Poland, but Bartkowska (1973) found it in Tatry Mountains (southern Poland). In turn, Skuratowicz (1967) indicates that this species occurs in Pomeranian region. Similarly, we reported a low similarity of flea species between central and eastern Poland (12 species/subspecies).

Aulak (1970) indicates that usually fauna of small mammals is richer in fertile and wet habitats. Thus, we supposed that a greater number of flea species could be recorded in fertile and wet habitats and lower in poor and arid habitats. The results of our study confirmed our predictions about the relationship between wetness of habitat and the number of flea species. However, this was not due to the increased number of host species or host abundance on richer habitats as we found no effect of habitat fertility or habitat wetness on number of small mammal species recorded or on small mammal abundance. The increased number of flea species recorded in more wet habitats could be due to the fact that humidity has a strong influence on the development of flea larvae (Skuratowicz 1967, Krasnov et al. 2006a) and relative humidity within burrows is one of the main factors influencing flea development and survival (Krasnov et al. 2001, Osacar-Jimenez et al. 2001). Therefore, more humid habitats could be suitable for bigger number of flea species than arid ones. On the other hand, we found no relationship between richness of habitat and the number of flea species.

Short term of our study undoubtedly had a negative impact on the number of captured mammals and collected fleas. It is probable that, with larger sample sizes and sampling in more localities, some of our predictions and observed trends would obtain stronger support. So, further studies are required to accurately determine the differentiation in flea species composition of small mammals in selected regions of Poland. Due to the fact that in Poland fleas spend the most of their time in host nests (Skuratowicz 1967; Krasnov et al. 2010a), fleas should be collected from both small mammals captured in live traps as well as from host nests. Additionally, it would be desirable to investigate more study plots in Pomerania region and highlands in order to increase the range of data allowing for determination of the impact of both latitude and longitude on flea communities.

## Appendix

**Table 4:** Description of the 19 study plots in the Słowiński National Park, Gorzowska Forest, Konin lakes area and in the Bieszczady Mountains, including Habitat richness and humidity, and trapping effort

Plot no.	Latitude and longitude	Plot description	Wetness	Richness	Trapping season	Number and layout of traps	Trapping effort [trap-hours]
Słowiński National Park							
S1	54° 41′ 59.74″ N, 17° 18′ 31.39″ E	Community similar to Pomeranian fertile beech forest *Melico-Fagetum* on moist soil; dominant plants: *Molinia cerulea, Fagus silvatica, Quercus sessilis*	2	1	July 2010	Grid: 3 parallel lines of 10 box traps	4,350
S2	54° 41′ 52.96″ N, 17° 18′ 47.83″ E	Peat alder forest *Sphagno squarrosi-Alnetum,* wet (water from 20 cm below ground surface to 20 cm deep); dominant plants: *Pteridium aquilinum*, *Molinia cerulea*, *Alnus glutinosa, Betula pubescens*	3	5	July 2010	Grid: 3 parallel lines of 10 box traps	2,700
S3	54° 41′ 40.00″ N, 17° 12′ 25.50″ E	Reeds *Phalaridetum arundinaceae*, wet (water 0–30 cm deep); dominant plants: *Phalaris arundinacea*, *Carex pseudocyperus*, *Glyceria maxima*	5	4	Aug 2010	Grid: 3 parallel lines of 10 box traps	1,185
S4	54° 42′ 4.95″ N, 17° 12′ 38.43″ E	Alder forest (shore of Dołgie Lake) and a mosaic of plant communities *Iridetum pseudacori* and *Cicuto-Caricetum pseudocyperi* (water 20–30 cm deep); dominant plants: *Carex pseudocyperus, Iris pseudoacorus, Carex nigra, Alnus glutinosa, Betula pubescens*	5	4	Aug 2010	Grid: 2 parallel lines of 15 box traps	1,125
S5	54° 40′ 17.39″ N, 17° 05′ 19.60″ E	Seaside pine forest *Empetro nigri-Pinetum*	3	4	Sep 2011	Grid: 3 parallel lines of 10 box traps	3,600
S6	54° 40′ 29.63″ N, 17° 03′ 55.27″ E	Wet meadow	4	2	Sep 2011	Grid: 3 parallel lines of 10 (14) box traps	3,600
S7	54° 40′ 23.92″ N, 17° 04′ 00.11″ E	White dune *Elymo*- *Ammophiletun arenariae*	1	1	Sep 2011	Grid: 2 parallel lines of 15 box traps	3,600
Gorzowska Forest							
G1	52° 41′ 22.18″ N, 15° 03′ 43.92″ E	Wet meadow with individual trees and shrubs and lush herbaceous vegetation (herbs and grass)	3	4	July 2010	Grid: 8 parallel lines of 8 box traps	36,864
G2	52° 41′ 58.14″ N, 15° 04′ 9.87″ E	Beech forest	2	2	July 2010	Grid: 8 parallel lines of 8 box traps	36,864
Konin lakes area							
K1	52° 18′ 9.17″ N, 18° 19′ 11.50″ E	Three microhabitats: lake bank with lush vegetation, herbs and bulrush; channel leading eutrophic water to a nearby wetland and ecotone between rather dry and wet meadow (southwestern bank of Licheńskie Lake)	3	5	Aug 2011	Grid: 3 parallel lines of 10 box traps	1,290
K2	52° 18′ 25.80″ N, 18° 18′ 21.98″ E	Dry aspect of alder forest separated with a causeway from the eastern bank of Pątnowskie Lake	1	1	Aug 2011	Grid: 3 parallel lines of 10 box traps	1,200
K3	52° 17′ 54.63″ N, 18° 11′ 42.22″ E	Two microhabitats: bank of a small forest lake with sedges and herbs, and moist alder forest (without standing water between the clumps) with dense stand (700 m west of Gosławskie Lake)	4	5	Aug 2011	Grid: 3 parallel lines of 10 box traps	600
K4	52° 17′ 43.48″ N, 18° 11′ 5.14″ E	Three microhabitats: forest lake bank overgrowing with the willow bushes and bulrush; wet (but without standing water between the clumps) alder forest and mixed forest on the side of the hill (500 m north of Skąpe Lake)	3	4	Aug 2011	Grid: 2 parallel lines of 10 box traps and a separate line (C) of 10 box traps	600
K5	52° 15′ 54.40″ N, 18° 15′ 25.93″ E	The bank of artificial and polluted lake including three microhabitats: steep bank overgrown with reeds, quite moist open plateau with blackberry and broom bushes and a steep slope overgrown with herbs and with a loose trees and grasses	3	3	October 2011	Grid: 3 parallel lines of 10 box traps	1,080
K6	52° 16′ 40.35′ N, 18° 16′ 14.44″ E	An anthropogenic and polluted environment (backwaters near the “Gosławice” Power); included three microhabitats: reeds growing on wetlands bank, sewer bank with lush rush and slope descending to this sewer covered with herbaceous vegetation with a few birches	4	3	October 2011	Grid: 3 parallel lines of 10 box traps	1,080
K7	52° 19′ 25.30″ N, 18° 21′ 7.98″ E	Three microhabitats: a large glacial lake bank with a narrow strip of riparian vegetation, steep slope covered with mature trees and the plateau with lush grassy places, old pines and younger oaks and maples (eastern bank of Licheńskie Lake)	2	2	October 2011	Grid: 3 parallel lines of 10 box traps	1,860
K8	52° 20′ 12.32″ N, 18° 21′ 28.07″ E	Two microhabitats: large glacial lake bank with a narrow strip of riparian vegetation with reeds and pine forest with herbaceous vegetation (northeastern bank of Licheńskie Lake)	2	2	October 2011	Grid: 3 parallel lines of 10 box traps	1,860
Bieszczady Mountains (in/near Lutowiska village)							
B1	49° 14′ 51.70″ N, 22° 40′ 14.84″ E	Pine forest with dense undergrowth dominated by blackberry	2	4	Aug 2011	Grid: 3 parallel lines of 10 box traps	2,070
B2	49° 15′ 0.77″ N, 22° 41′ 24.67″ E	Banks of a stream running through wet meadow with individual shrubs and lush herbaceous vegetation (reeds and herbs)	4	3	Aug 2011	Grid: 3 parallel lines of 10 box traps	1,680

**Table 5:** Species and numbers of all small mammals captured in selected habitats of the Baltic coast, the central lowlands, and mountains of Poland

		Słowiński National Park							Gorzowska Forest		Konin lakes area								Bieszczady Mountains	
Mammal species	Code	S1	S2	S3	S4	S5	S6	S7	G1	G2	K1	K2	K3	K4	K5	K6	K7	K8	B1	B2
*Apodemus agrarius*	Agr	–	–	1	1	–	1	3	–	–	19	–	2	2	14	28	5	4	–	5
*Apodemus flavicollis*	Afl	4	16	2	1	–	–	1	49	31	–	1	4	9	14	1	3	1	10	23
*Apodemus sylvaticus*	Asl	–	–	–	–	–	–	–	–	–	–	–	–	–	4	–	–	–	–	–
*Micromys minutus*	Mmn	–	–	–	–	1	12	–	–	–	–	–	–	–	–	–	–	–	–	–
*Microtus agrestis*	Mgr	–	–	–	–	9	–	–	–	–	–	–	–	–	–	–	–	–	–	1
*Microtus arvalis*	Mar	–	–	–	–	–	–	–	–	–	–	–	–	–	–	–	–	–	–	5
*Microtus oeconomus*	Moe	–	1	10	11	–	4	–	–	–	–	–	–	–	–	–	–	–	–	–
*Myodes glareolus*	Mgl	–	3	–	–	1	–	–	44	11	12	3	23	4	11	4	–	–	14	–
*Neomys fodiens*	Nfd	–	–	–	1	–	–	–	–	–	2	–	1	1	–	2	–	–	–	2
*Sorex araneus*	Sar	–	1	1	1	1	–	–	–	–	2	1	1	1	1	6	1	–	3	11
*Sorex minutus*	Smn	–	1	2	3	–	1	–	–	–	–	–	–	–	–	–	–	–	–	1
Total		4	22	16	18	12	18	4	93	42	35	5	31	17	44	41	9	5	27	48
